# Supplementary material for: Are carbon emissions trading and green financial instruments synergistic? -Comprehensive quantitative research based on content analysis
Source: PLoS One. 2024 Mar 7;19(3):e0298601. doi: 10.1371/journal.pone.0298601 (PMC10919601; doi:10.1371/journal.pone.0298601)
Supplement: S1 Data — (ZIP) [file pone.0298601.s001.zip › Data/地级市Z_F工作报告.zip!/╣π╬≈/║╪╓▌╩╨/└┤╫╘╣1⁄2╓┌║┼í╛┬φ┐╦╩2╛▌═°í┐_2011.docx]

各位代表:

　 　现在,我代表贺州市第二届人民政府向大会作工作报告,请予审议,并请市政协委员和其他列席人员提出意见。

一、过去五年的工作回顾

　　 市二届人大一次会议以来的五年,是贺州发展不平凡的五年。面对国际金融危机严重冲击和雨雪冰冻、旱涝等自然灾害严重影响,我们在自治区党委、政府和市委的坚强领导下,在市人大及其常委会、市政协的监督支持下,以科学发展观为统领,主动顺应国家宏观调控,努力化解发展中的困难和矛盾,全面实现了“十一五”规划的目标任务,圆满完成了市二届人大一次会议确定的各项工作任务,开创了科学发展跨越发展新局面。

　 　──经济持续快速发展,综合实力明显增强。地区生产总值由2005年的141.1亿元增加到2010年的296.7亿元,年均增长12%；人均生产总值由6703元提高到14580元。财政收入由11.2亿元增加到22.1亿元,年均增长14.5%。全社会固定资产投资由83亿元增加到363.4亿元,年均增长34.3%。三次产业结构由36.3:34.4:29.3优化为21.5:47.0:31.5,工业主导型经济基本形成。地区生产总值、工业增加值、城镇居民人均可支配收入等14项主要经济指标均实现翻番。

　 　──支柱产业培育壮大,工业主导地位强化。累计完成工业投资425亿元,年均增长37.2%。工业增加值由2005年的35.4亿元提高到2010年的107.7亿元,年均增长14.8%。工业对经济增长的贡献率提高到50%。电力、林产、矿业、电子和新材料五大支柱产业逐步发展壮大；食品、制药、建材、农产品加工等传统优势产业竞争力不断提升。贺州华润循环经济示范区、电子科技生态产业园、旺高工业区、西湾(平桂)工业区、信都经济区、桂台(贺州)客家文化旅游合作示范区等六大产业园区规划建设加快推进。平桂飞碟、桂东电子、嘉宝食品等一批原有骨干企业发展壮大,金源稀土、吉光电子、新凯骅木业等一批新引进工业项目建成投产,经济发展的产业支撑能力明显增强。桂东电子、金源稀土、灵峰药业等3家企业被认定为国家级高新技术企业,工业产品技术含量有新提高。

　 　──循环经济加快发展,发展模式初具雏形。编制实施《贺州市循环经济发展规划》、《广西贺州华润循环经济示范区发展规划》,大力发展循环经济成为我市转变经济发展方式的有效途径。全面深化与华润集团战略合作,共同规划建设贺州华润循环经济示范区,扎实做好创建全国循环经济示范区各项基础性工作。示范区核心区内总投资8亿元的华润富川水泥一期工程建成投产,总投资78亿元的华润贺州电厂一期项目建设加快推进,总投资3亿元的华润雪花啤酒一期项目启动建设,“三厂一区”循环经济发展模式初具雏形。随着华润水泥、电力、啤酒等项目落户贺州,“央企入贺”有了新突破,开辟了产业发展和循环经济园区建设的新路子。

　 　──城镇化步伐加快,城乡面貌日新月异。城建项目累计完成投资247亿元,城镇化率由2005年的30%提高到2010年的35%。完成修编《贺州市城市总体规划(2009—2030年)》、《“一江两岸三新区”控制性详细规划》,控制性详细规划覆盖率从30%提高到80%。完成城区农民建房及产业用地规划,制定出台《贺州市城市规划区范围内建设项目规划和用地审批管理办法》等一系列政策措施,城市建设管理逐步规范。新设立平桂管理区,城市发展空间进一步拓展。启动实施涉及49个项目、总投资达187亿元的三年城市建设大会战,建成贺州火车站、新客运中心、污水处理厂、垃圾填埋场和火车站站前大道、南环路、光明大道、东环路等项目；规划建设市文化中心、太白湖公园、绿洲家园、火车站至电子科技生态产业园大道、西南环路、灵峰大桥等项目,“一江两岸三新区”路网工程等基础设施建设全面推进,城市功能与品质明显提升,先后荣获自治区市容“南珠杯”竞赛第六届特等奖、第七届优秀城市奖。全面完成城乡风貌改造和农村危房改造任务。启动重点镇村产业发展规划编制,加强重点镇村建设,小城镇发展活力和承载能力明显增强。

　　──现代农业提质增效,农村经济全面发展。农业综合生产水平明显提高,粮食实现连年增产,农林牧渔业总产值由2005年的65亿元提高到2010年的100亿元,年均增长4.6%。农业结构不断调整优化,茶叶、脐橙、马蹄、瘦肉型猪、优质家禽等优势农业向规模化、特色化、品牌化发展,先后有12个农产品获国家级、自治区级名优农产品称号。农业产业化经营加快推进,雨润集团、广东温氏等农业龙头企业落户贺州,市级以上重点农业产业化龙头企业由2005年的11家发展到2010年的36家,直接带动农户扩大到20.9万户。在全区率先启动创建出口食品农产品质量安全示范区工作,建成农业标准化生产基地面积50万亩；建立无公害标准化养殖示范场141个；通过自治区无公害农产品产地认证面积98.3万亩；建成出口备案基地17个,面积12.8万亩。新农村建设稳步推进,组织建设新农村示范点135个,农村道路、水利、教育、文化、卫生等基础设施建设步伐加快。

　　──商贸旅游业巩固提高,第三产业快速发展。服务业增加值由2005年的41.3亿元增加到2010年的90.9亿元,年均增长13.3%。全社会消费品零售总额由34.5亿元增加到78.7亿元,年均增长18.3%。商贸基础设施不断完善,远东国际城、上海街、香港城、金旗市场、湖广大市场等项目建设稳步推进。商贸流通业繁荣稳定,房地产业有序开发。金融业加快发展,2010年末全市金融机构存款余额比2005年末增长1.7倍,贷款余额增长2.4倍。保险、信息咨询、商务中介、社区服务等新兴服务业加快发展。旅游业稳步发展,成功创建“中国优秀旅游城市”。围绕打造华南生态旅游名城和国际知名的客家文化旅游目的地,重点规划建设桂台(贺州)客家文化旅游合作示范区,姑婆山客家风情小镇、贺州五星级酒店等项目开工建设,成功举办桂台客属联谊会暨贺州市首届客家文化旅游节,精心打造姑婆山、黄姚古镇、十八水等一批旅游品牌、精品景区和特色线路,先后创立国家4A级景区3个、3A级景区1个。黄姚古镇获“中国历史文化名镇”称号,秀水状元村获“中国历史文化名村”称号。全市旅游接待总人数累计达1926.6万人次,比“十五”期间增长96.3%；旅游总收入119.7亿元,增长198.9%。

　 　──基础设施日臻完善,发展基础不断夯实。水利电力基础设施条件明显改善,累计争取上级资金　9.7亿元,实现投资36.8亿元,完成56座病险水库除险加固等多项设施维护和建设任务,恢复灌溉面积12.6万亩,改善灌溉面积19万亩。交通发展全面加快,实施两轮总投资超300亿元的基础设施建设大会战,累计完成投资244.7亿元,建成铁路130公里、高速公路198.4公里,新建、改建农村道路2447.8公里,在全区率先实现全部乡镇通等级油路或水泥路的目标。洛湛铁路、桂梧高速公路和广贺高速公路贺州段建成通车,改写了我市没有铁路和高速公路的历史。贵广高速铁路、永贺高速公路贺州段开工建设,贺州经黄姚至昭平二级公路即将全线通车,贺州火车站至姑婆山、富川至望高二级公路改造工程正在施工,大通道网络将进一步完善,交通末梢正在向区域性枢纽转变。

　 　──项目建设加快推进,投资拉动作用明显。围绕基础设施、产业发展、社会公益等领域,采取非常办法、非常措施、非常力度和非常政策,扎实推进重点项目建设,“六个一”项目推进模式得到自治区的充分肯定并在全区推广。全社会固定资产投资累计完成1016.8亿元,是“十五”期间的5.7倍,其中亿元以上重大项目180项、总投资748亿元,已完成投资379亿元,投资对经济发展发挥了强劲拉动作用。

　 　──改革开放不断深化,发展活力日益增强。扎实推进重点领域和关键环节体制机制改革。金融体制改革步伐加快,整合组建贺投、城投、交投和旅投四大投融资公司；“引银入贺”取得新成果,引进设立了桂林银行贺州支行；商业银行升格工作稳步推进,建行、农行、工行和中行四大国有银行先后升格为分行或区分行直管支行；设立小额贷款公司6家、融资性担保公司3家。财税体制改革不断深化,在全区率先实施房地产税一体化征收管理改革,财源培植和税收征管工作进一步加强。粮食购销企业产权、农村集体林权、土地流转等各项制度改革稳步推进。对外开放不断扩大,招商引资工作成效明显,累计引进项目2630个,到位资金721.7亿元；完成进出口总额5.8亿美元,年均增长15.4%。

　　──环境保护力度加大,生态建设取得实效。组织实施了新一轮土地利用和矿产资源总体规划。严格执行耕地保护法,保持耕地总量动态平衡。大力开展植树造林,加强生态公益林、自然保护区建设,实施退耕还林、珠江防护林、农村沼气等重点生态工程建设,全市森林覆盖率达到70.4%；累计建设沼气池16.1万座,沼气入户率达到57.6%,连续五年居全区前列。强力推进节能减排,全面完成自治区下达的“十一五”节能减排和淘汰落后产能任务,重点行业、企业能耗水平和排污强度实现较大幅度下降。建成污水处理厂5个、垃圾填埋场4个,中心城区污水集中处理率达到80%,各县城达到60%,生活垃圾无害化处理率均达到100%。持续开展重点河流、湖库、矿山和城镇农村环境综合治理,“贺江清新”行动取得新进展。

　　──人民生活有效改善,和谐社会创新发展。城乡居民收入持续提高。城镇居民人均可支配收入由2005年的7516元增加到2010年的15802元,年均增长15.4%。农民人均纯收入由2351元增加到4298元,年均增长12.8%。各级财政在教育、卫生、文化、社会保障和就业等民生领域累计投入87.5亿元,比“十五”时期增长175.5%。“小财政办大民政”经验得到国家民政部的充分肯定。全面完成141个贫困村“整村推进”扶贫开发任务,投入各项财政扶贫资金1.45亿元,减少贫困人口15.2万人,解决45.7万人饮水安全问题,解决8.4万人用电困难。加强城乡就业工作,新增城镇就业7.3万人,转移农村劳动力20.4万人,城镇登记失业率控制在4.5%以内。城镇居民基本医疗保险和新型农村社会养老保险试点工作进展顺利,城乡低保实现“应保尽保,按标施保”目标。新建保障性住房8732套,建筑面积81.9万平方米,低收入家庭住房条件不断改善。“五保村”建设项目获第三届中国地方政府创新奖,被国家民政部树立为“一面旗帜”。扎实推进社会和谐稳定模范市创建工作,深入开展平安贺州建设,不断强化社会治安综合治理,社会大局和谐稳定。安全生产和食品药品监管工作不断加强。国防教育和双拥共建活动成效显著,荣获“自治区双拥模范城”、“全国双拥模范城”称号。

　　──社会建设全面加强,各项事业协调发展。科技创新取得新进展,实施第三、第四轮创新计划项目408项,引进消化吸收创新工业先进技术44项,集成创新和推广应用农业先进实用技术86项,参与制订国家和行业标准10项,启动并推进厅市会商制度,全面实施知识产权发展战略,专利申请量、授权量大幅度提升。教育事业优先发展,实现城乡免费义务教育,“两基”工作顺利通过“国检”；农村中小学校布局调整扎实推进,城区教育项目建设大会战启动实施,全市自治区级示范性普通高中达到6所,新建市幼儿园已开园办学；职教攻坚顺利通过自治区达标验收,5所中等职业学校迁入市职教中心办学；贺州学院在校生超过1万人。文化事业开创新局面,文明和谐创建活动广泛开展,荣获“自治区文明城市”称号；积极实施文化惠民工程,建设村级公共服务中心64个,农家书屋202个,有29个乡镇357个村配备了文化活动设施；“村村通”广播覆盖率达94%,电视覆盖率达98.1%；特色文化影响力扩大,成功举办中国(贺州)瑶族盘王节；临贺故城、马殷庙被列入国家级文物保护单位,拥有4项国家级非物质文化遗产,客家山歌剧《仙姑岭茶歌》和瑶族蝴蝶歌《流水欢歌迎客来》荣获国家社会文化最高奖──全国第十五届“群星奖”。卫生事业实现新突破,新一轮医药卫生体制改革取得阶段性成效,公共卫生和医疗服务体系更加健全,医疗保障能力进一步增强,新农合参合率提高到94.3%；市人民医院门诊综合楼、市妇幼保健院住院楼和各县人民医院综合楼加快建设,各级医疗卫生基础设施不断改善；加强重点传染病防控工作,重大疾病预防控制成效明显。人口计生工作迈上新台阶,持续稳定低生育水平,人口自然增长率控制在8‰以内；“诚信计生”工作得到国家和自治区计生委的充分肯定,连续五年被评为全区人口计生工作先进单位,先后被确定为全国婚育新风进万家活动示范市、全区首批人口计生综合改革示范市,在全区率先实现“全国计划生育优质服务先进县(区)”全覆盖。全民健身公共服务体系不断完善,竞技体育取得新成绩。统计、物价、人力资源、机构编制、外事侨务、民族宗教、住房公积金、工会、供销合作、工商、税务、质量技术监督、检验检疫、防震减灾、气象、库区移民、档案、史志、烟草、驻梧管理、决策咨询等工作取得新进展,妇女儿童、老龄、残疾人等事业实现较快发展。

　　──政府建设扎实推进,公共服务职能强化。圆满完成“五五”普法任务。坚持政府常务会议学法制度,推进依法行政,建设法治政府。认真贯彻落实市委决策,自觉接受人大、政协及各民主党派、工商联和社会各界的监督,人大代表议案建议、政协委员提案办结率均达到100%。完成新一轮市、县政府机构改革。加快政府职能转变和管理创新,取消行政审批事项212项,下放审批权限127项。扎实推进公共资源交易监管体制改革,自2007年在全区率先启用市级公共资源交易平台以来,全市采购招标交易项目3138项,节约资金7.5亿元。建立政府新闻发言人制度,政府信息公开力度不断加大。坚持实施为民办实事工程,着力解决群众关心的热点、难点问题,群众生产生活条件不断改善。建立完善市、县、乡三级政府应急管理体系,应急管理工作逐步迈上正轨。全面加强行政监察和审计工作,深入推进党风廉政建设和反腐败工作,查处了一批违法违纪案件,提高了政府的执行力和公信力。

　 　五年的发展实践,我们深切地体会到,加快贺州改革发展,必须始终坚持在发展中不断深化对市情的认识,不断创新和完善后发展欠发达地区推动科学发展跨越发展的思路,积极应对复杂局面,有效破解发展难题,努力在调整中加快发展、在竞争中跨越发展；必须始终坚持把大力发展循环经济作为转变经济发展方式的突破口和主攻方向,促进产业转型升级,形成更加突出的后发优势,努力实现贺州后发崛起；必须始终坚持大投入大建设,不断优化投资方向、加快投资速度、提高投资效率,不断培育新的经济增长点,努力依靠优质增量做大经济总量；必须始终坚持实施东靠战略不动摇,牢固树立强烈的开放带动意识,主动接轨“珠三角”,积极承接东部产业转移,加快向先进生产力靠拢,努力增强发展活力、拓展发展空间；必须始终坚持以人为本,把政府公共资源更多转向民生,从解决事关人民群众切身利益的现实问题入手,着力保障和改善民生,努力让人民群众共享发展成果；必须始终坚持转变政府职能,强化作风建设,狠抓工作落实,努力推动经济社会又好又快发展。

　 　各位代表,过去的五年,是我市基础增强、提速发展的五年。回顾过去,成绩来之不易,这是自治区党委、政府和市委正确领导的结果,是全市各族人民团结奋斗、顽强拼搏的结果,是市人大、市政协和社会各界有效监督、大力支持的结果。在此,我谨代表市人民政府,向努力工作在各条战线上的广大干部群众,向各位人大代表、政协委员、离退休老同志,向各民主党派、工商联、无党派人士、各人民团体,向驻贺部队、武警官兵,向来贺创业的投资者和劳动者,向所有关心支持贺州发展的社会各界人士,表示衷心的感谢并致以崇高的敬意!

　 　在充分肯定成绩的同时,我们也清醒地认识到,我市后发展欠发达地区的基本市情没有根本改变,经济社会发展中还存在不少困难和问题,主要表现在:经济总量偏小,人均水平较低；产业结构不优,自主创新能力不强；基础设施不完善,公共服务保障能力不足；中心城市辐射带动能力不强,城镇化水平有待进一步提高；改革开放深度广度还不够,一些体制性障碍尚待破解,土地、资金、能源等要素制约依然突出；政府职能需要进一步转变,服务意识、执行水平需要进一步提高。对此,我们将高度重视,认真研究,采取切实有效的措施加以解决。

二、今后五年的工作任务

　　 今后五年,是我市科学发展跨越发展的加速期、转型发展的攻坚期、富民惠民的提升期

　　 根据市第三次党代会提出的“两年有新突破,五年上新台阶,十年实现新跨越”总体目,发展的机遇和挑战并存,机遇大于挑战。世界多极化和经济全球化深入发展,经济格局进行深度调整,国际产业转移和区域经济合作进一步深化；我国转变经济发展方式和调整经济结构步伐加快,各方面体制机制不断完善,社会政治大局稳定,国际国内环境总体上有利于我们加快发展。随着国家新一轮西部大开发、《国务院关于进一步促进广西经济社会发展的若干意见》和广西“富民强桂”新跨越战略的深入实施,广西桂东承接产业转移示范区、桂台(贺州)客家文化旅游合作示范区加快建设,大通道网络进一步完善,增添了贺州科学发展跨越发展的外源动力。经过“十一五”的努力,我市经济保持着良好的发展势头,综合实力明显提升,发展思路更加清晰,并积累了新经验,营造了良好环境,加快发展的内生动力不断增强。特别是当前我市进入了工业化城镇化加速发展的关键时期,有着不可替代的后发优势。与此同时,我们也面临着各种严峻挑战。国际金融危机给世界经济造成深度冲击,当前欧美债务危机持续恶化,国际形势依然复杂多变,将直接影响中国未来的发展。国内发展中仍然存在不少矛盾和问题,特别是深层次市场化改革滞后,结构调整和发展方式转变难度还很大。受经济大环境和自身发展水平等诸多因素影响,我市面临着后发展欠发达地区加快发展的任务越来越重、向前追赶的压力越来越大、人民群众追求美好生活的诉求越来越强等方面挑战考验。纵观大局,我们面临的机遇与挑战都是前所未有的,只有牢牢把握各种有利机遇,积极应对各种挑战,全力破解各类难题,才能在新的历史起点上推动我市更好更快发展。

　 　根据市第三次党代会提出的“两年有新突破,五年上新台阶,十年实现新跨越”总体目标,确定今后五年政府工作的主要任务是:以邓小平理论和“三个代表”重要思想为指导,深入贯彻落实科学发展观,全面落实市第三次党代会的决策部署,认真实施“工业立贺、富民强市”战略,按照把我市建设成为“全国循环经济示范区、广西新兴工业城市、桂粤湘区域性交通枢纽、华南生态旅游名城”的发展定位,加快转变经济发展方式,大力发展循环经济,不断深化改革开放,加强基础设施建设,统筹推进城乡发展,切实保障和改善民生,全面完成“十二五”规划的目标任务,努力开创科学发展跨越发展新局面,建设富裕文明和谐幸福新贺州。

　 　今后五年,我市经济社会发展的主要目标是:

　 　──综合实力显著增强。到2012年贺州建市十周年时,经济社会发展良好势头进一步巩固和扩大,地区生产总值突破400亿元,财政收入突破30亿元,全社会固定资产投资突破600亿元,工业总产值突破520亿元。到2015年,在全面实现“十二五”规划目标的基础上,力争实现地区生产总值600亿元,财政收入50亿元,全社会固定资产投资800亿元,工业总产值1000亿元,均比2010年翻一番。

　 　──城乡发展更加协调。到2012年,以“一江两岸三新区”为核心的城市发展格局初步形成,中心城市建成区面积达到34平方公里,城镇化率达到38%；县域经济规模不断壮大,小城镇发展水平明显提高,新农村建设快速推进,城乡差距进一步缩小,向世人初步展现一个崛起的崭新贺州。到2015年,中心城区建成区面积达到40平方公里,城镇化率达到48%,市政设施不断完善,城市管理水平明显提升,桂粤湘区域性交通枢纽初步形成。

　 　──社会环境更加和谐。到2015年,社会管理水平不断提升,民主法制进一步健全,社会更加和谐稳定,社会事业更加协调发展。生态环境更加优美,森林覆盖率达到72%以上,城镇污水处理率达到85%,垃圾无害化处理率达到100%,全面实现“十二五”节能减排刚性目标,循环经济体系初步建立,初步形成资源节约型、环境友好型社会。

　 　──人民生活更加富裕。到2012年,城镇居民人均可支配收入达到19100元,农民人均纯收入达到5400元。到2015年,城镇居民人均可支配收入达到25800元,农民人均纯收入达到7500元。低收入群体收入明显增加,中等收入群体持续扩大,人民群众拥有更多财产性收入,享有更多基本公共服务,幸福指数明显提升。

　 　为实现上述目标,着重加快推进以下十个方面工作:

　 　(一)加快推进新型工业化,巩固提高工业主导地位。深入实施“工业立贺”战略,着力建设“五大支柱产业,六大产业园区,一条工业走廊”,加快推进新型工业化进程,建设广西新兴工业城市。

　 　打造五大百亿元产业。在改造升级传统产业的基础上,把发展的触角延伸到高新技术产业和先进制造业,全力打造电力、林产、矿业、电子、新材料等五大支柱产业集群,形成对经济发展的重要支撑。加快推进华润贺州电厂建设,不断扩大桂东电力的产业优势,加强与南方、华中等大电网合作,构建完善的区域供电网络,推动电力产业集群发展。充分发挥森林资源优势,加快推进林桨纸一体化项目,发展造纸、板材、家具制品,形成林产林化循环经济产业链。加大矿产资源整合力度,建立集勘探、采选、冶炼、研发、加工和综合利用开发体系,努力打造有色金属、建材等矿业产业集群,加快把资源优势转化为经济优势。加快建设广西电子铝产品质量监督检验中心,大力引进铝电解电容器生产企业集群,重点推进桂东电子中高压电子铝箔技改、桂海铝业铝光箔、吉光电子和江浩电子高档电容器等项目建设,促进电子铝箔产业向上延伸到精铝、铝光箔生产环节,向下延伸到相关铝电子产品生产环节,推进电子、电器产业集群发展,打造全国铝电子产业基地。加快发展稀土功能材料和钨钛锡等新材料产业。到2015年,力争电力产值达到100亿元、林产产值达到100亿元、矿业产值达到250亿元、电子产值达到200亿元、新材料产值达到100亿元以上,五大支柱产业产值占规模工业总产值比重70%以上。

　　抓好产业园区和工业走廊建设。坚持走产业集聚、要素集约、布局优化、特色鲜明的园区经济发展道路。创新园区建设模式,主动引进外资、支持民间资本投入园区开发建设。加强与大企业、大集团和战略投资者的合作,支持采取园中园、共管园、托管园等多种模式,建立利益分享机制,合作共建、代建和运营工业园区。高起点、高标准、高品位推进特色产业园区建设,重点加快建设贺州华润循环经济示范区、电子科技生态产业园、旺高工业区、西湾(平桂)工业区、信都经济区、桂台(贺州)客家文化旅游合作示范区等六大特色产业园区,提升产业发展承载能力。培育形成以华润电厂、水泥、啤酒为核心的循环经济产业链,力争把贺州华润循环经济示范区建成全国循环经济示范区。充分发挥贺州投资集团的优势作用,在确保江浩电子、翔隆电子、桂海铝业等企业入园发展的基础上,吸引更多关联企业投资,努力把电子科技生态产业园建成全国铝电子产业基地。进一步加强与中国铝业、中国有色等大型央企合作,努力把旺高工业区建成集稀土功能材料、钨钛锡新材料以及食品、药品于一体的特色园区和国家战略性新材料产业高地,把新材料工业园建成在区内外有一定影响力的高新技术产业园区。积极引进中国通用技术集团等大企业、大集团,开发利用丰富的大理石资源,努力把西湾(平桂)工业区建成全国重要的石材粉体深加工基地。立足毗邻广东的区位优势,充分利用信都经济区、电子科技生态产业园等园区基础条件,密切与佛山市的合作,联合规划、共同建设粤桂合作贺州佛山开发试验区,推动“飞地”经济发展,积极承接东部转移产业。同时,依托207、323国道和广贺高速公路,建设信都至富川的百里工业走廊,加快构建我市重要的战略产业带。按照“同类企业整合做大做强,不同类企业实施错位发展”的原则,不断调整优化百里工业走廊的产业结构和布局,形成分工合理、梯度互补的产业体系,构建大通道经济发展新格局。到2015年,力争百里工业走廊实现产值850亿元,占全市工业总产值80%以上。

　　加快发展中小企业和强优企业。深入实施“千家中小企业成长工程”、“企业家成长和产业工人成才促进计划”。通过产业引导、政策配套、资金扶持、环境优化、培训服务等措施,培育发展一批中小企业和微型企业,发展壮大一批拥有自主知识产权和知名品牌的强优企业,锻炼一支高素质产业工人队伍。加强经济运行监测监控,强化煤电油运等生产要素保障,引导企业趋利避害健康发展。到2015年,力争新增工业企业600家、年产值亿元以上企业60家；有5家以上优质企业进入上市培育辅导期,2家以上企业实现上市。

　 　(二)加快推进城镇化,建设科学合理的城镇体系。坚持把推进城镇化与工业化有机结合起来,促进产业园区与城市新区互动发展,强化科学规划和扩城提质,提高城镇经营管理水平,加快建设桂粤湘区域性中心城市。

　 　加快城镇规划体系建设。以科学规划引领城镇建设,进一步完善城乡规划,加强贺州城区、县城以及重点镇规划与总体规划无缝衔接,加快形成城乡一体化的规划体系。加快‘贺八平钟一体化’进程,加速形成以贺州城区为中心,钟山县城为副中心,富川、昭平县城为卫星城的城镇整体布局。大力推进中心城区扩容提质,将黄田、沙田、鹅塘、莲塘、贺街纳入城区建设管理框架,逐步实现与主城区“同城化”发展。

　　抓好中心城区建设。以迎接建市十周年为契机,深入实施城市建设大会战,统筹推进城东新区、江南新区、火车站新区等新城区建设。创新城市建设运营模式,多渠道筹措资金,加快建设新城区路网、供水排污管网,完善餐饮、购物、休闲娱乐等商业服务设施,拓宽新城区框架,完善新城区服务功能。加快推进市文化中心、太白湖公园、绿洲家园、贺州五星级酒店等标志性重点工程建设,加快形成“一江两岸三新区”发展新格局。优先发展公共交通,加快建设市政道路、管道燃气、防洪工程、供电供水、文化教育、科技、医疗卫生等公共服务设施,提升城市综合承载能力。

　 　加快县城和重点镇建设。加快三县县城、重点镇和名镇名村建设,促进产业、人口向城镇集聚。钟山县城以全面融入市区发展为目标,努力建设成为贺州副中心城市；富川县城以建设“中国瑶乡明珠”为目标,充分彰显民族特色,利用生态品牌发展成为宜居宜业县城；昭平县城以建设宜居休闲型山水森林城为目标,依托良好的生态环境和秀美风光,打造以休闲旅游为特色的山水生态县城。坚持城镇带动、重大产业带动和园区带动,集中推动重点城镇群发展。突出抓好百里工业走廊沿途乡镇基础设施建设,增强城镇为工业发展服务的功能,实现工业化城镇化互动发展。抓好城乡风貌改造工程和生态文明新农村建设示范点工作,加快昭平县黄姚镇、富川瑶族自治县秀水村和福溪村等特色名镇名村建设,促进城镇化和新农村建设良性互动。

　 　创新城镇经营管理机制。健全“两级政府、分级管理”的城镇管理体系,加大集镇和中心村庄规划、建设、管理力度,提高中心城镇的带动作用。加强城市规划管理,研究制定老城区改造管理实施办法,完善新城区建设管理规定。强化土地节约集约利用管理,加大土地整理和储备工作力度,建立以招标、拍卖、挂牌出让为主要形式的土地供应机制。强化土地卫片执法检查,加大违法建筑、违法占地治理力度。完善现行户籍、教育、医疗、社保、就业、住房等政策制度,逐步消除制约农民工市民化的体制性障碍,使农民工享有基本公共服务,实现安居乐业。深化完善城乡清洁工程长效管理机制,强化市容环境综合整治,提高城镇绿化、美化、亮化、净化水平。

　　(三)加快推进现代农业发展,全面建设社会主义新农村。按照城乡一体化要求,全面落实支持县域经济发展政策措施,着力建设具有产业支撑的新农村,促进农业增效、农民增收、农村发展,加快建设桂粤湘区域性特色农产品基地和物流集散中心。

　 　加快农业产业化步伐。坚持用抓工业的理念来发展现代农业。以建设特色、高效、生态、品牌农业为重点,加快建设蔬菜、水果、茶叶、生猪等标准化绿色农产品生产基地,建成广西首个出口食品农产品质量安全示范区。深入实施万元增收工程、“234”农业产业化工程和三大增粮增收行动,加快推进贺州福润200万头生猪加工、广东温氏、贺州巨东等一批农业产业化项目,大力发展农业生产经营服务组织和农民专业合作组织。到2015年,力争实施生态农业及绿色食品加工项目30个,发展市级以上重点农业产业化龙头企业50家,农业生产组织化程度达到60%以上。

　 　完善现代农业服务体系。有效整合各项涉农资金,加快农产品地方标准、动植物疫病防控和保护、农产品质量安全检验检测、农业技术和农机推广服务、农产品流通等公共服务体系建设。大力发展订单农业、网上销售、直销配送等新型营销模式,进一步拓宽农产品销售渠道。完善现有水果、蔬菜批发市场的设施建设,加快金泰农产品加工物流中心、富川桂湘粤农产品加工物流中心建设步伐。到2015年,力争全市大中型农产品批发市场达到5家,农产品年交易量达到180万吨以上。

　 　扎实推进新农村建设。突出抓好新农村示范点建设,精心打造“十镇百村百里文明走廊”,因地制宜建设一批名镇名村。推进农村道路、农田水利、农村电网以及文化站、图书室、乡镇卫生院等公共基础设施建设,健全农村公共服务体系,改善农村生产生活条件。推进农村危房改造和水库移民新村建设。全面落实各项惠农强农政策,促进农民持续增收。抓好各类技能培训,提高农民职业技能和创业创收能力。加大扶贫攻坚力度,着力提高农村贫困人口收入。

　 　发展壮大县域经济。实施工业强县和项目带动战略,因地制宜发展壮大县域特色主导产业,做大做强工业集中区,提高工业在县域经济中的比重。加快城镇化进程,综合运用各项扶持政策,推动人口集聚、功能集聚、产业集聚,使城镇成为县域经济发展的重要平台和增长点。充分利用资源优势,着力打造“一乡一业”、“一村一品”,搞好农副产品深加工,促进农业产业化发展。用好用活国家促进民族地区、山区发展和自治区实行“省直管县”财政改革、加快工业化城镇化等一系列优惠政策和保障机制,吸引外资,激活民间资本参与发展壮大县域经济。依托区位优势、资源优势和产业优势,努力把八步区建设成为承接东部产业转移的桥头堡、陶瓷生产基地、林产工业强区和现代农业示范区；把钟山县建设成为桂东北机械制造和矿产资源加工基地、特色农业示范县；把富川瑶族自治县建设成为循环工业强县、电力工业强县、特色优势农业强县和瑶乡文化旅游目的地；把昭平县建设成为林产工业大县、生态休闲旅游名县和茶叶产业强县；在统筹推进平桂管理区申请正式设区工作的基础上,把平桂建设成为现代化新城区和战略性新兴产业基地。　　(四)加快推进现代服务业发展,促进产业结构优化升级。坚持市场化、产业化和社会化方向,进一步改善服务业基础设施,优化服务业结构,突出发展商贸物流业和生态旅游业,加快建设区域性商贸物流基地和华南生态旅游名城。

　 　大力发展商贸物流业。优化城市商业结构和空间布局,加强农贸市场规划建设,重点建设一批服装、建材、汽配、五金、家电、农副产品等特色商业街、专业批发市场和交易中心,建设亿丰(贺州)国际商贸城,营造商业集聚区,提升城市商业服务功能与水平。扩大对外贸易,以培育出口主体和增强企业自营出口能力为重点,扩大加工贸易出口规模,促进外贸持续快速发展。到2015年,力争出口总额达1.5亿美元,年均增长12%,年出口额超3000万美元的企业达5家。加快发展现代物流业,加大仓储配送、运输装卸、货物代理、联合运输等物流业基础设施项目建设。引进北部湾国际港务集团投资建设贺州无水港区物流中心,加快建设雨润农产品物流中心,培育一批物流龙头企业。合理布局物流网点,大力发展综合物流园区、专业物流中心和物流配送中心,建设市火车站仓储区、西湾南部工业仓储区、马鹿坪工业仓储区等一批仓储基地,实现物流运输顺畅对接。加快物流组织系统和公共信息平台建设,积极发展连锁经营、配送、代理等新型流通业,促进第三方物流和电子商务物流发展。

　 　着力发展生态旅游业。整合优质旅游资源,精心打造姑婆山生态旅游区、大桂山旅游休闲度假区、桂江生态旅游景区、黄姚古镇、临贺故城、客家围屋、富川秀水状元村和钟山十里画廊等重点旅游景区景点,加快旅游产品改造升级。提升一批乡村农业旅游示范点,推动城乡旅游产业联动发展。充分挖掘丰富的自然旅游资源、民族文化风情资源以及客家文化资源,把桂台(贺州)客家文化旅游合作示范区建成广西与台湾文化旅游交流合作的窗口、广西接待台湾游客的主要旅游目的地。加强旅游区域合作,加快融入大桂林旅游圈,主动与“泛珠三角”地区对接,开拓旅游客源市场,实现旅游接待总人数年均增长12%、旅游总收入年均增长16%以上的目标,着力把我市打造成集生态观光、运动健身、养生休闲、历史考古、民族历史文化考察、民俗人文考究和文化拓展于一体的华南生态旅游名城。

　　积极发展各类服务业。加快发展信息网络、科技、服务外包等生产性服务业。有序繁荣休闲娱乐、体育健身、养老保健和社区服务、物业管理、家政服务等生活服务业。大力发展信息咨询、职业中介、经纪代理、营销策划、咨询评估、产权交易等中介服务业。提升发展酒店、餐饮业。积极稳妥发展房地产业,针对升级换代的刚性需求,引进有实力的房地产企业,对城区进行有序开发和改造,打造环境优美、管理服务规范的中、高档舒适小区,配套建设中小户型的保障性住房。大力发展银行、保险、证券等金融业,继续加快“引银入贺”步伐,鼓励、吸引国内外金融机构在贺州开设分支机构。推动和完善各类新型金融组织体系建设,发展多层次的贷款担保基金和担保机构,鼓励和支持设立村镇银行、小额贷款公司、农村资金互助合作社等新型金融机构,为中小企业和农业发展提供资金保障。

　 　(五)加快推进基础设施建设,提升支撑发展能力。加强交通、能源、信息、水利基础设施建设,加快构建布局合理、设施先进、功能齐全、城乡共享的基础设施体系。

　 　完善综合交通运输体系。着力在提高交通密度、速度和通达程度上下功夫,加快构建“五高三铁两江一机场”立体交通网络。重点是加快推进贵广高速铁路和永贺高速公路贺州段建设,加快推进柳韶铁路、洛湛铁路复线及其电气化改造工程,贺州至韶关、贺州至巴马、信都至梧州高速公路,贺江扩能、桂江航道工程和贺州民用机场等重大交通项目前期工作,争取早日开工建设,加快形成桂粤湘区域性交通枢纽,融入广州两小时经济圈和北部湾三小时经济圈。力争规划建设贺街至富阳一级公路,建成贺州火车站至姑婆山二级公路,实现主要产业园区、旅游景区景点等重要节点与铁路、高速公路、国道、省道无缝连接。

　 　加强能源基础设施建设。以加强电源建设、完善供配电网络、发展清洁能源为重点,打造广西重要的能源基地。加大电网基础设施建设力度,争取华润贺州电厂一期项目在2012年建成投产,新建、扩建一批变电站和输电线路,建成全区重要的“西电东送”能源基地和中转站。抓住国家实施新一轮农村电网改造升级工程的机遇,加大农村供电设施项目建设力度,改善农村供电条件。大力发展清洁能源,推进富川龙头、长春、金子岭风电场和钟山唱歌山风电场等风电项目,以及旺高工业区太阳能光伏发电项目建设。加快城市天然气管网建设,建设“西气东输”梧州至贺州支线和主要工业园区燃气管网铺设工程,建立安全、清洁、高效的能源供应体系。

　 　加大水利设施建设力度。紧紧抓住水利建设黄金十年的历史机遇,以民生水利、惠农水利、生态水利建设为重点,全面实施江河治理、饮水安全保障、病险水库除险加固和农田水利建设四大会战。到2015年,力争建成中心城区“一江两岸”和昭平、钟山、富川县城防洪堤工程,开展贺江、桂江干流沿途重要乡镇防洪治理,完成16条中小河流治理任务；全面完成病险水库除险加固任务；启动实施路花水库水源工程,解决中心城区缺少备用水源问题；有效解决83万农村人口饮水不安全问题；继续推进龟石大型灌区续建配套与节水改造建设,着重开展合面狮、花山、龙潭、鸟源等4个骨干中型灌区建设；实施小农水重点县建设,力争新增农田有效灌溉面积29.8万亩。

　 　加快信息化基础设施建设。布局建设新一代移动通信、互联网、数字电视等网络设施及宽带无线城市、农村宽带网络,构建城乡信息基础设施网络。推动电信、数字电视、互联网“三网融合”,构建宽带融合安全的信息基础设施。加快电子政务、公共信息服务网络平台和电子商务交换平台建设,扩大信息技术在经济社会发展各领域的应用,大力推动信息化与工业化城镇化的深度融合,着力打造“数字贺州”。

　 　(六)加快推进项目建设,发挥投资强劲拉动作用。高度重视项目建设,不断优化投资结构,形成基础设施和产业投资并举的投资新格局,充分发挥投资对扩大内需、推动发展的重要作用。

　 　科学谋划项目。按照国家产业政策、宏观调控方向和中央投资重点领域,及时谋划一批影响我市发展大局的重点项目、民间资金参与建设的中小项目和政府主导的社会事业项目,建立完善动态项目储备库,形成“谋划一批、开工一批、投产一批”滚动推进局面。加快推进“十二五”规划重点项目前期工作,争取国家和自治区安排的重大产业项目、基础设施项目在我市多布局快建设,着重加大新兴工业、特色农业、现代服务业以及交通、能源、水利、城镇基础设施等领域的投资力度。

　 　完善重点项目推进机制。继续坚持和完善“六个一”项目推进机制,继续实行重大项目联合审批制度,强化项目跟踪督查、协调服务和监督管理,着力解决项目推进中的困难和问题,实行严格的绩效考核和奖惩问责制度,全力推进重点项目建设,促进项目尽快开工建设、竣工达产,确保我市“十二五”规划的重大项目顺利推进。

　 　着力破解项目制约问题。加快信用担保体系建设,发展融资担保公司；深化银企合作,促进企业、项目业主与金融机构对接,解决项目建设资金问题。充分发挥国有融资公司的作用,拓展银行信贷融资主渠道。探索投资主体多元化、融资方式多样化、运作方式市场化的项目建设新机制,综合运用BT、BOT等方式,努力扩大项目融资规模。统筹抓好项目用地保障工作,积极争取国家、自治区给予更多用地指标支持,推进城乡建设用地增减挂钩工作,缓解用地指标不足的压力；坚持节约集约用地,加大盘活存量土地力度；做到依法征地,和谐拆迁,确保项目建设用地落实到位。

　 　(七)加快推进改革开放,聚集加快发展要素。进一步深化各个领域的改革,加快形成更加有利于经济发展方式转变、推动科学发展跨越发展的机制体制,全面实施东靠战略,主动融入多区域合作,进一步增强发展活力。

　 　全面推进各项改革。深化财政管理改革,加强公共预算体系建设,不断提高财政科学化、精细化、法制化管理水平。大力培植财源,增强财政筹措资金和保障正常运转的能力。优化支出结构,设立工业发展基金,加大财政对支柱产业发展的支持力度。强化信用意识,加强政府债务管理,建立健全债务清偿和债务风险预警机制,增强抵御和化解债务风险的能力。深化投融资管理体制改革,加强对国有投融资公司的指导,促进投融资公司健康、规范、有序发展。加快推进农村产权改革,深化集体林权制度改革,完善农村土地流转机制。加快电网整合,理顺电力管理体制,积极争取“直供电”政策,进一步优化电力供应结构。

　 　不断深化区域合作。积极拓展与“珠三角”、闽苏浙、港澳台之间的区域合作,主动接受先进生产力的辐射带动,主动融入北部湾。借助中国-东盟自由贸易区平台,拓展与东盟国家在经贸、旅游等领域的合作。深化与广东佛山、清远、韶关、肇庆和湖南永州等市际合作与交流,重点加快粤桂合作框架内的贺州佛山开发试验区规划建设。完善桂东四市联席会议等合作机制,争取中央、自治区加大对广西桂东承接产业转移示范区的政策、项目和资金扶持。积极参与西江经济带建设,推动贺江、桂江流域经济社会发展。加快与东部地区及周边城市的交通对接、产业对接和市场对接,不断扩大招商引资和承接产业转移工作成效。创新招商引资方式,继续推进产业招商、园区招商、以商招商,着力引进世界500强、中国500强企业落户贺州。深入开展招商引资项目大兑现活动,加强对引进项目的跟踪指导服务,进一步提高项目履约率、资金到位率和开竣工率。

　 　大力发展非公有制经济。进一步完善和落实鼓励非公有制经济发展的政策措施,支持中小企业做大做强,大力发展微型企业。加快建立中小企业信用评价体系,健全融资担保体系,促进金融机构加大对非公有制企业信贷投放力度。拓展非公有制经济发展空间,鼓励和引导民间资本进入基础产业、基础设施、公共服务、社会事业、保障性住房建设等领域。

　 　(八)加快推进生态文明建设,建设环境美好家园。大力发展循环经济,加强生态环境保护,加大节能减排力度,加快创建国家森林城市和广西园林城市、生态文明示范城市步伐。

　 　加快发展循环经济。按照“政府主导、市场推进、公众参与”原则,在企业、园区和社会三个层面同时推进循环经济发展,加快构筑循环型工业、循环型农业和循环型社会体系。积极探索新型资源开发利用和工业发展模式,形成一批循环型工业企业和产业园区。重点发展电力、建材、有色金属、稀土及钨、生物制药、林产、食品深加工等循环经济产业,打造电力-建材、电力-轻工-制药、啤酒-生化-食品-养殖、铝-电深加工、石材及其副产品深加工等循环产业链。着力把六大产业园建设成为循环经济园区,推动区域循环经济发展。围绕新农村建设,大力推广以沼气为纽带的农业循环经济发展模式,重点打造养殖-沼气-种植、农林产品加工及资源化利用等循环产业链。引导全民参与,倡导绿色消费,发展绿色社区,建设绿色政府,大力开展全社会节水、节能、节地行动,重点加快发展特色循环型旅游业等现代服务业。

　　 加强生态环境保护。加快林业生态工程建设,继续实施山区生态林、重点区域防护林、自然保护区建设和小流域水土保持综合治理等重点生态工程,扎实推进姑婆山、滑水冲、七冲等生态文明基地建设。深入开展“绿满贺州”造林绿化行动,全面实施通道绿化、城镇绿化和村屯绿化工程,力争完成造林绿化115万亩。加强水资源管理和水生态建设,实施水土保持治理、水源地保护、水生态修复工程,实现水资源的可持续利用。深入推进“贺江清新”行动,加强贺江、桂江重点流域和水库水污染防治力度。强化环境风险和地质灾害防范,进一步巩固地质灾害治理成果。加强工业污染防控工作,强化矿山地质环境保护与治理恢复,加强矿山开采秩序管理。积极探索农村垃圾处理新模式,不断加强农村环境综合治理,切实改善农村生态和人居环境。

　 　开展节能减排攻坚。强化新开工项目节能评估和环评把关,从源头上加强能源节约和污染防控。积极推进重点行业和重点企业节能降耗,坚决淘汰落后产能,提高能源利用水平。全面推广清洁生产,完善污染防治体系。深入开展企业节能低碳、节能减排和绿色建筑全民行动。严格落实目标责任制,督促企业完善污染治理设施,抓好重点污染源监控,依法查处治理设施不运行和违法排污行为。完善节能减排指标监测和考核体系,继续推进主要污染物总量减排,全面完成“十二五”节能减排目标任务。

　　(九)加快推进以民生为重点的社会建设,营造和谐稳定环境。坚持以人为本,切实解决群众最关心、最直接、最现实的利益问题,不断满足人民群众日益增长的物质文化需求,提高人民群众的幸福指数,促进社会和谐稳定。

　　促进就业再就业。做好高校毕业生、农村转移劳动力、退役军人和随军家属等群体的就业工作。全面落实全民创业政策,支持创办微型企业,帮助高校毕业生、返乡农民工等各类人员通过创业实现就业。适度发展劳动密集型产业,以大项目实施扩大就业。积极开发就业岗位,改善就业结构,扩大就业规模。强化职业技能培训,提高失业人员创业能力和再就业能力。

　 　努力增加居民收入。合理调整收入分配关系,统筹制定覆盖社会各阶层的增收政策措施,努力提高居民收入在国民收入分配中的比重,使群众实际收入与经济发展水平基本相适应。坚持实行最低工资标准,逐步提高扶贫标准,保障低收入者的基本生活。完善征地补偿安置制度,使农民更多地分享农业用地转为非农业用地过程形成的土地增值收益。加快集体林权制度改革和林业发展步伐,提高农民各项补偿收入。全面落实粮食直补、退耕还林补助等各项补贴政策,使农民增加国家政策性补贴收入。

　 　提高社会保障水平。强化政府住房保障职责,通过新建、改建、收购、租赁等方式,大力实施安居工程。加大保障性住房建设和城乡危旧房、棚户区改造力度,积极推进驻梧管理处机关大院危旧房改造,建立完善多层次保障性住房体系。到2015年,力争建设经济适用房10万平方米,新增廉租房6万平方米。健全覆盖城乡居民的基本医疗保障体系,继续落实基本养老、医疗保险制度,完善失业、工伤、生育保险制度和城乡最低生活保障制度,实现新型农村社会养老保险制度全覆盖。完善农村“五保”集中供养模式。完善救灾救济等社会救助体系。采取积极应对措施,加强市场价格监测预警和监督检查,努力控制物价过快上涨势头,保持物价总体水平基本稳定。

　 　推进社会管理创新。创新社会管理机制,健全维护群众权益机制,努力构建和谐劳动关系。深入开展“和谐建设在基层”和社会矛盾“大排查、大接访、大调处、大防控”等主题活动,巩固提高信访维稳工作成果。启动实施“六五”普法规划,不断推进依法治市进程。深入开展平安贺州建设,完善社会治安动态防控体系,提高人民群众的安全感。加强防灾、抗灾、减灾和救灾体系建设。推进国防和民兵预备役建设。高度重视公共安全,进一步完善市、县、乡三级政府应急体系,加强应急救援队伍、应急平台建设,强化应急物资储备,提高应对公共突发事件的预测、预警、预报和处置能力。强化食品药品监管,保障公众食品用药安全。认真落实安全生产企业主体责任和政府监管责任,努力保持安全生产形势持续稳定,加快建设社会和谐稳定模范市。

　 　重视科技、教育和人才工作。加快推进第五轮科技创新计划。积极推动产业技术创新,进一步加大政府对科研的投入,鼓励企业增加技改资金,逐年提高全社会科研和发展经费支出占GDP的比重,逐步建立以企业为主体、市场为导向、产学研结合的技术创新体系。加大科技研发力度,应用高新技术和先进适用技术改造提升传统产业,推进产业结构优化升级,重点扶持发展光铝电子、新材料、医药等产业,培育拥有自主知识产权的重大产业科技项目。加大科技惠农力度,重视民生科技。全面实施知识产权发展战略,力争专利申请量年均增长25%以上,科技进步对经济增长的贡献率达到40%以上。坚持教育优先发展,促进各类教育协调发展,进一步巩固“两基”达标成果,深入实施国家中长期教育改革和发展规划纲要,启动实施“学前教育三年行动计划”。到2015年,实现每个乡镇建有公办幼儿园,30%以上行政村有公办幼儿园(班)。全面落实义务教育经费保障机制,大力调整学校布局,促进义务教育均衡发展。规划建设市青少年活动中心,完成贺州高中新校区建设、贺州第二高中扩建任务,加快普及高中阶段教育,大力完善现代职业教育体系。扶持发展高等教育,加快推进贺州学院新校区二期工程建设,大力推进与地方主导产业相吻合的学科和专业建设。继续实施人才强市战略,办好各类引才引智活动,拓宽人才资源开发与交流合作渠道,努力引进高层次人才和急需专门人才；建立完善科学的人才评价、服务、激励制度,在全社会形成尊重人才、关心人才、重视人才的良好氛围,充分发挥人才在经济社会发展中的作用。

　　全面提升人口计生工作水平。加大统筹人口计生工作力度,创新开展“诚信计生”,积极构建“幸福计生”,巩固稳定低生育水平。提升计划生育优质服务水平,提高出生人口素质,降低出生缺陷发生率,遏制出生人口性别结构失衡。加强人口和计划生育公共服务体系建设,完善利益导向机制,让实行计划生育家庭得到更多实惠。深化村(居)民自治,推进人口计生工作法制化、民主化管理。加强流动人口服务管理,实现计划生育基本公共服务均等化。

　 　加快文化、体育和卫生等事业发展。把社会主义核心价值体系建设融入文化建设的全过程,深化群众性精神文明创建活动,提高市民素质和城市文明程度。积极开展新一轮“全国双拥模范城”创建活动,广泛开展形式多样的群众性双拥文化宣传活动和双拥共建活动。加强和改进未成年人思想道德建设,促进未成年人全面发展。加大公益文化设施建设投入,健全公共文化服务体系,着力推进市文化中心、图书馆、群众艺术馆、博物馆、档案馆、妇女儿童活动中心、科技馆等文化项目建设,建设一批村级公共服务中心,实现县(区)有文化馆、图书馆、数字影院,乡镇有文化站,村村有“农家书屋”。加快发展影视制作、演艺娱乐、出版印刷、工艺美术、文化创意等文化产业,加强重点文物、物质文化遗产地和非物质文化遗产保护传承。大力弘扬传统文化,打造“瑶族盘王节”、“客家文化旅游节”等特色文化品牌,着力建设以瑶族文化、客家文化、民俗文化为特色的民族文化生态保护区。实施文化精品工程,打造一批艺术精品。加强体育人才培养,加快公共体育设施建设,广泛开展全民健身活动,提升竞技体育水平,加快发展体育产业。全面深化医药卫生体制改革,进一步提高新农合保障水平,加快公共卫生和医疗服务体系建设,推进村级卫生室建设,促进基本公共卫生服务均等化,全面推进防治艾滋病攻坚工程。加快殡葬事业改革发展。继续做好统计、审计、机构编制、档案、地方志、民族宗教、外事侨务、住房公积金、工会、供销合作、工商、税务、检验检疫、质量技术监督、库区移民、气象、防震减灾、驻梧管理等方面工作。

　 　(十)加快推进政府自身建设,不断提高公共服务水平。建设富裕文明和谐幸福新贺州,对政府自身建设提出了更高的要求。要进一步转变政府职能,推进管理创新,努力建设法治型、效能型、服务型政府。

　 　转变政府职能。切实发挥经济调节、市场监管、社会管理和公共服务职能。正确履行经济调节职能,综合运用经济、法律和行政手段推动结构调整和发展方式转变；加强市场监管职能,强化对涉及人民群众生命财产安全领域的监管；更加注重社会管理职能,深入推进社会矛盾化解和社会管理创新；更加注重公共服务职能,加强公共基础设施建设,健全公共财政政策,完善公共服务体系,提高公共服务质量。

　 　严格依法行政。认真执行人大及其常委会的决议和决定,自觉接受人大及其常委会的法律监督、工作监督和政协的民主监督,认真办理人大代表议案、建议和政协委员提案,广泛听取各民主党派、工商联、无党派人士和人民群众意见,支持工会、共青团、妇联等群众团体开展工作。完善政府工作规则,坚持依法科学民主决策,强化行政监督和行政问责,落实行政执法责任制,进一步增强市、县两级政府的决策能力、财政能力、廉政能力。深化政务公开,重点推进财政预算、公共资源配置、重大建设项目、公益事业等领域政务信息公开,切实保障群众的知情权、参与权和表达权。加大政府信息公开力度,加强电子政务建设,重视舆论监督和网络监督。全面落实廉政建设责任制,严格执行部门预算、政府采购等制度,加强对重点领域、关键部位、特殊岗位的监督管理,发挥监察、审计的监督作用,从源头上预防腐败。

　　加强作风建设。简化办事程序,精简会议文件,改进会风文风,提高工作效率。切实厉行节约,勤俭办事,降低行政成本。坚持问政于民、问需于民、问计于民,广泛开展调查研究,使政府工作思路、工作部署、政策措施更加符合民意。健全机关效能监察机制,严肃查处政令不畅、办事效率低下、行政不作为等问题。完善目标管理绩效考核机制,认真落实行政问责制,进一步提高工作执行力。

　 　各位代表,贺州正站在新的历史起点上,新的征程任重道远,美好前景催人奋进。让我们在自治区党委、政府和市委的正确领导下,深入贯彻落实科学发展观,凝聚全市人民的智慧和力量,以更加宽广的视野、更加饱满的热情、更加扎实的工作,为建设富裕文明和谐幸福新贺州而努力奋斗!
